# Efficient open cultivation of cyanidialean red algae in acidified seawater

**DOI:** 10.1038/s41598-020-70398-z

**Published:** 2020-08-24

**Authors:** Shunsuke Hirooka, Reiko Tomita, Takayuki Fujiwara, Mio Ohnuma, Haruko Kuroiwa, Tsuneyoshi Kuroiwa, Shin-ya Miyagishima

**Affiliations:** 1 Department of Gene Function and Phenomics, National Institute of Genetics, 1111 Yata, Mishima, Shizuoka 411-8540 Japan; 2 Department of Genetics, Graduate University for Advanced Studies (SOKENDAI), 1111 Yata, Mishima, Shizuoka 411-8540 Japan; 3 National Institute of Technology (KOSEN), Hiroshima College, 4272-1 Higashino, Osakikamijima, Toyota, Hiroshima 725-0231 Japan; 4 Department of Chemical and Biological Science, Faculty of Science, Japan Women’s University, 2-8-1 Mejirodai, Bunkyo-ku, Tokyo, 112-8681 Japan

**Keywords:** Physiology, Plant sciences

## Abstract

Microalgae possess high potential for producing pigments, antioxidants, and lipophilic compounds for industrial applications. However, their open pond cultures are often contaminated by other undesirable organisms, including their predators. In addition, the cost of using freshwater is relatively high, which limits the location and scale of cultivation compared with using seawater. It was previously shown that *Cyanidium caldarium* and *Galdieria sulphuraria,* but not *Cyanidioschyzon merolae* grew in media containing NaCl at a concentration equivalent to seawater. We found that the preculture of *C. merolae* in the presence of a moderate NaCl concentration enabled the cells to grow in the seawater-based medium. The cultivation of cyanidialean red algae in the seawater-based medium did not require additional pH buffering chemicals. In addition, the combination of seawater and acidic conditions reduced the risk of contamination by other organisms in the nonsterile open culture of *C. merolae* more efficiently than the acidic condition alone.

## Introduction

Microalgae are a highly diverse group of photosynthetic organisms found in both marine and freshwater habitats. They have the ability to produce many beneficial products, such as pigments, antioxidants, and lipophilic compounds for industrial applications^1^. However, industrial applications of microalgae are limited to the production of relatively expensive materials because of the high costs associated with their cultivation. Open ponds are the simplest systems for mass algal production and cost less to build and operate than closed photobioreactors^2^. However, open pond systems are often unstable because they are easily contaminated by other undesirable microorganisms, especially predators of the cultivated algae^3^. Thus, the successful cases have been limited to a few algal species and involved cultivating an extremophilic alga in a particular environment that is lethal for many other organisms. For example, the alkaliphilic cyanobacteria *Spirulina* spp. are cultivated with a high concentration of bicarbonate and at a high pH^4^. Additionally, the halophilic green alga *Dunaliella salina* is cultivated in high saline water^4^. These algae are currently used for the production of cosmetics, nutraceuticals, and food coloring^5^.

Another issue related to the costs for algal cultivation is that the preparation of freshwater is costly and there are limited locations available for algal cultivation, which requires larger amounts of water than conventional crops. In addition, the United Nations predicts that by 2025 more than half of the countries in the world will be experiencing freshwater stress or outright shortages^6^. Thus, the use of seawater instead of freshwater is desired to reduce the cost and expand the scale of microalgal cultures. For example, *Spirulina* spp*.,* which inhabits alkaline freshwater lakes, can be cultivated in seawater supplemented with an inorganic nitrogen source, such as phosphate, bicarbonate, and Fe-EDTA^7^, and this seawater-based cultivation has been adopted by some companies^8^. In addition, a recent study showed that the freshwater model cyanobacterium *Synechocystis* sp. PCC6803 also grew in artificial seawater supplemented with nitrogen and phosphorus sources^9^.

The freshwater cyanidialean red algae, which are unicellular and include three recognized genera, *Cyanidioschyzon*, *Cyanidium*, and *Galdieria*, dominate in sulfuric acidic hot springs worldwide (pH 0.05–5.0, 35–56 °C). Thus far, the genomes of three species have been sequenced^10-13^. Recently, several attempts to use cyanidiales for industrial applications have been performed based on the following features of these algae.

Cyanidiales grow at a very low pH, which reduces the risk of contamination by other organisms. Cyanidiales possess phycocyanin, which is more thermo- and acid-stable than that of *Spirulina*^14-16^. *Galdieria* spp. can be used to recover rare metals^17^ and remove organic carbon and nutrients^18^ from various wastewaters. Among cyanidiales, *Cyanidium* spp. and *Galdieria* spp. are tolerant to a high salinity that is equivalent to seawater^19^. Thus, it is likely feasible to culture these species in an acidified seawater. In addition, because there is no highly acidic seawater in nature, and therefore no organism has been evolutionarily adapted to such environment, the combination of acidic water and seawater probably reduces the risk of contamination by other organisms more efficiently than acidic water alone. In contrast, it was previously reported that *Cyanidioschyzon merolae* was not able to grow in the presence of NaCl at the concentration equivalent to natural seawater^20^. However, *C. merolae* has the following features that are potentially useful for industrial applications, which are not present in *Cyanidium* spp. and *Galdieria* spp.

Similar to many other microalgae, the cells of *Cyanidium* spp. and *Galdieria* spp. are enclosed by a rigid cell wall^19^ that requires mechanical processing to be disrupted to release their cellular contents. In contrast, *C. merolae* does not possess a rigid cell wall making it is easier to extract cellular contents, for example, by drying, hypotonic treatment, or neutralization^21^. In addition, unlike other microalgae that are currently used for industrial uses, *C. merolae* is genetically tractable, and their transformants stably express transgenes without any gene silencing^21^. Furthermore, *C. merolae* can be genetically modified by self-cloning^21^.

Given the above information and assumptions, here we show that *C. merolae*, *Cyanidium caldarium*, and *Galdieria sulphuraria* can be cultured in natural seawater supplemented with inorganic nitrogen and phosphorus sources, iron, and a trace metal mix. In the case of *C. merolae*, the preculture of the cells in a moderate concentration of NaCl resulted in cellular growth in the seawater-based medium. We also show that the acidified seawater reduces the risks of microbial contamination in outdoor open cultivation. Because acidophilic freshwater algae have been identified in many other eukaryotic lineages, the combination of acid and seawater are likely also useful for the open cultivation of other microalgae to reduce the risk of contamination and costs associated with their cultivation.

## Results and discussion

### Limits of NaCl concentration for the growth of cyanidialean red algae

**Figure 1:**
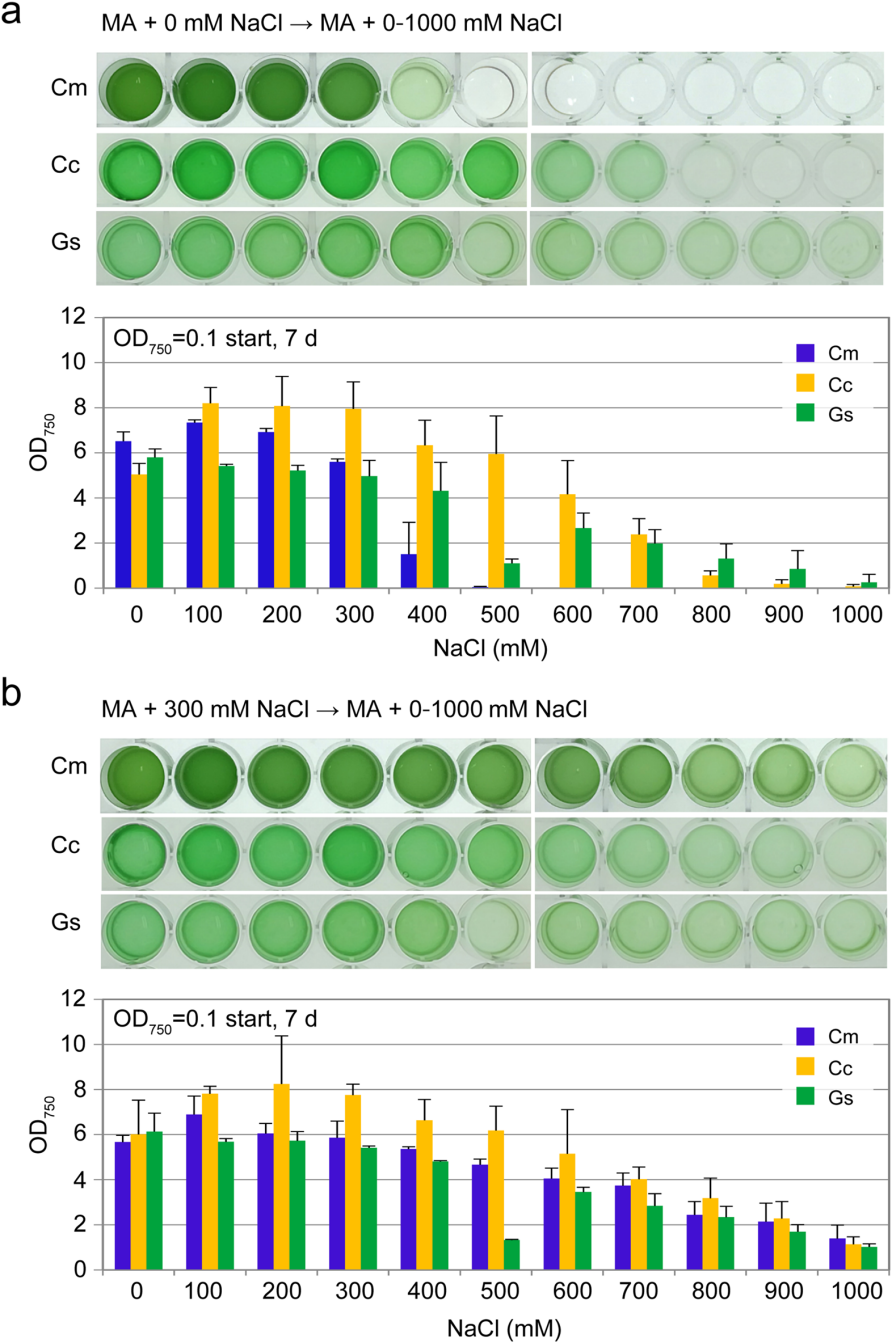
Growth of *C. merolae* 10D, *Cy. caldarium* RK-1, and *G. sulphuraria* 074 W in the inorganic MA medium supplemented with different concentrations of NaCl (0–1000 mM). (**a**) A photograph and OD_750_ of the cultures 7 days after inoculation. Cells cultured in MA (OD_750_ of 1.0–2.0) without additional NaCl were collected by centrifugation and resuspended in the respective media to give an OD_750_ of 0.1 and then cultured for 7 days in 24-well plates. Each data point represents the average and the error bar represents the standard deviation of three independent experiments. (**b**) A photograph and OD_750_ of cultures 7 days after inoculation. Cells cultured in MA with 0.3 M NaCl (OD_750_ of 1.0–2.0) were collected by centrifugation, resuspended in the respective media to give an OD_750_ of 0.1, and then cultured for 7 days. Each data point represents the average and the error bar represents the standard deviation of three independent experiments. Cm, *C. merolae* 10D; Cc, *Cy. caldarium* RK-1; Gs, *G. sulphuraria* 074 W.

As a first step to develop a cultivation system of cyanidiales in acidified seawater, we examined whether the three cyanidialean red algae *C. merolae* 10D, *Cy. caldarium* RK-1, and *G. sulphuraria* 074 W grew in an inorganic medium containing a concentration of NaCl that was equivalent to the amount in seawater (on average, the salinity of seawater is approximately 600 mM) and under our culture conditions. To determine the limits of NaCl concentration, the three algal strains that were grown in MA medium^22^ (a freshwater acidic medium) were transferred to MA medium supplemented with different concentrations of NaCl (from 0 to 1000 mM) and cultured (Fig. 1a). *Cy. caldarium* and *G. sulphuraria* were able to grow in media that contained ≥ 600 mM NaCl, which is consistent with a previous study, although the cultivation medium and conditions were different from the ones used in this study^19^. The growth rate of *G. sulphuraria* at 500 mM NaCl was lower compared with 400 or 600 mM. Although this result was reproducible, the mechanism contributing to these differences is unclear at this point. In contrast to *Cy. caldarium* and *G. sulphuraria, C. merolae* did not grow in the medium containing ≥ 500 mM NaCl. This result is also consistent with a previous study, although the cultivation medium and conditions were different compared with the ones used in this study^20^.

In general, the exposure of an organism to a particular stress leads to the acclimation of the organisms to that specific stress in a time-dependent manner^23^. Thus, we tested whether exposure of the three algal strains to moderate salt stress could increase their salt tolerance. To this end, the cells that were grown in MA with 300 mM NaCl were transferred to the media supplemented with different concentrations of NaCl (from 0 to 1000 mM) and cultured (Fig. 1b). We observed that the pre-cultivation in MA with 300 mM NaCl expanded the limit of NaCl concentrations for growth of the algal species, and *C. merolae* became tolerant to NaCl above 600 mM, which was equivalent to seawater (Fig. 1b).

### Cultivation of *C. merolae* in natural seawater-based media

As described above, we successfully prepared *C. merolae* cells that grew in the presence of NaCl at a concentration equal to or above seawater. However, this result does not necessarily indicate that the cells are able to grow in seawater-based media because certain components of seawater other than NaCl likely inhibit cellular growth. Thus, we also tested whether *C. merolae* cells precultured in MA medium with 300 mM NaCl could grow in the natural seawater-based medium.

**Figure 2:**
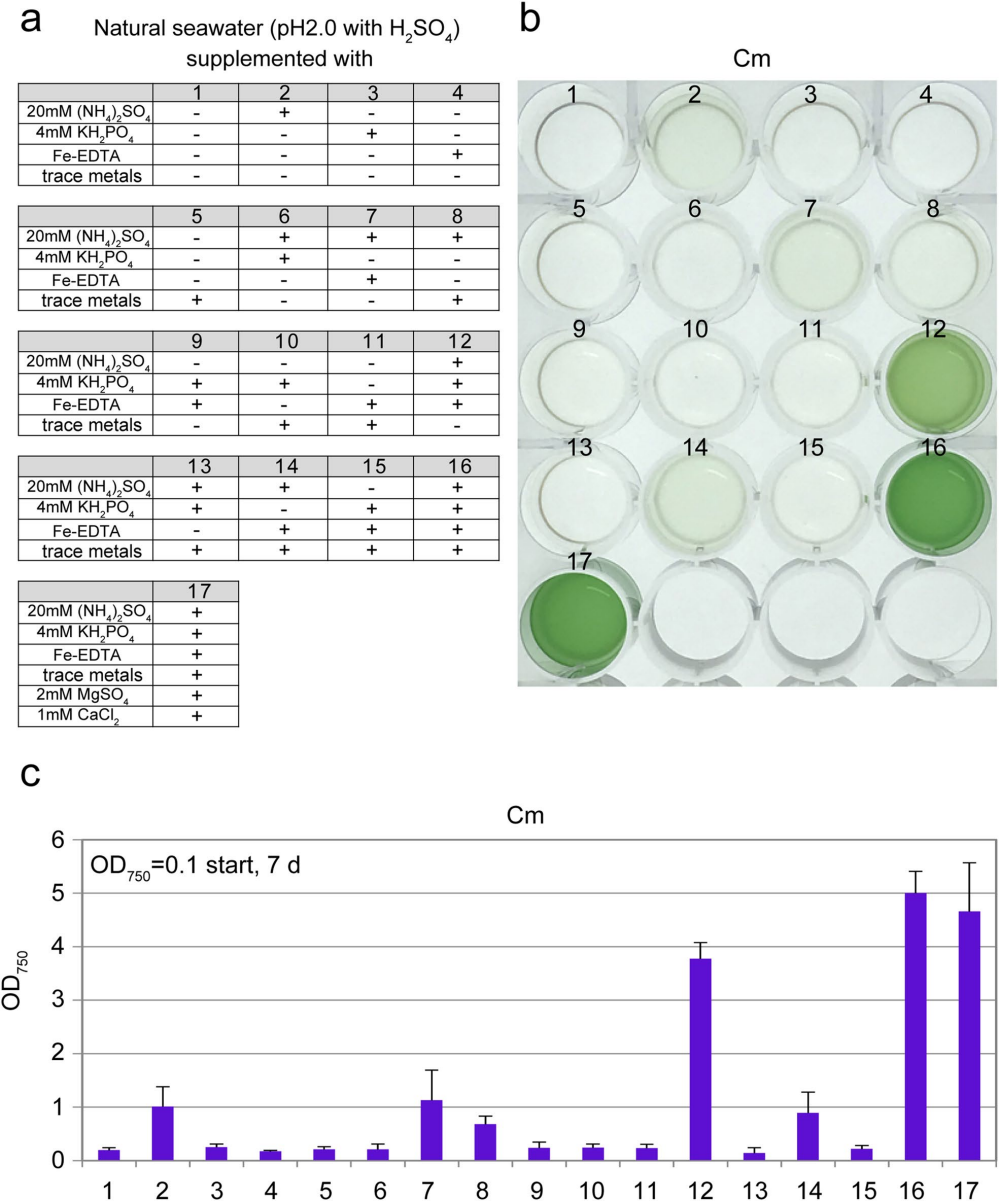
Growth of *C. merolae* in the acidified natural seawater supplemented with different inorganic nutrients. (**a**) Composition of respective media. (**b**) Cells cultured in MA with 0.3 M NaCl (OD_750_ of 1.0–2.0) were collected by centrifugation, resuspended in the respective media to give an OD_750_ of 0.1, and then cultured for 7 days. The photograph shows the cultures in the respective media 7 days after inoculation into a 24-well plate. (**c**) OD_750_ of the cultures 7 days after inoculation. Each data point represents the average and the error bar represents the standard deviation of three independent experiments.

The synthetic inorganic MA medium (pH2.0), which is suitable for the growth of cyanidialean red algae, contains (NH_4_)_2_SO_4_, KH_2_PO_4_, MgSO_4_, CaCl_2_, Fe-EDTA, and a trace metal mix^22^. According to the chemical composition of the medium, natural seawater, which was adjusted to pH 2.0 with H_2_SO_4_, was supplemented with several combinations of (NH_4_)_2_SO_4_, KH_2_PO_4_, Fe-EDTA, and trace metals, in which the concentration of each inorganic chemical was the same as in the MA medium. In natural seawater supplemented with all of the above chemicals, *C. merolae* cells grew to give an OD_750_ of approximately 5 in 7 days (Fig. 2c, #17), which was comparable to the cells cultured in MA supplemented with 600 mM NaCl (Fig. 1b). Furthermore, we found that additional MgSO_4_ or CaCl_2_ did not affect the growth rate in the presence of (NH_4_)_2_SO_4_, KH_2_PO_4_, Fe-EDTA, and a trace metal mix (Fig. 2c, #16). In contrast, the absence of either (NH_4_)_2_SO_4_, KH_2_PO_4_, Fe-EDTA, or a trace metal mix reduced or abolished *C. merolae* growth (Fig. 2c). These results showed that the acidified natural seawater supplemented with (NH_4_)_2_SO_4_, KH_2_PO_4_, Fe-EDTA, and the trace metal mix supports *C. merolae* growth, which is comparable to the synthetic MA medium.

### Growth of the cyanidialean red algae in the presence of different nitrogen sources

**Figure 3:**
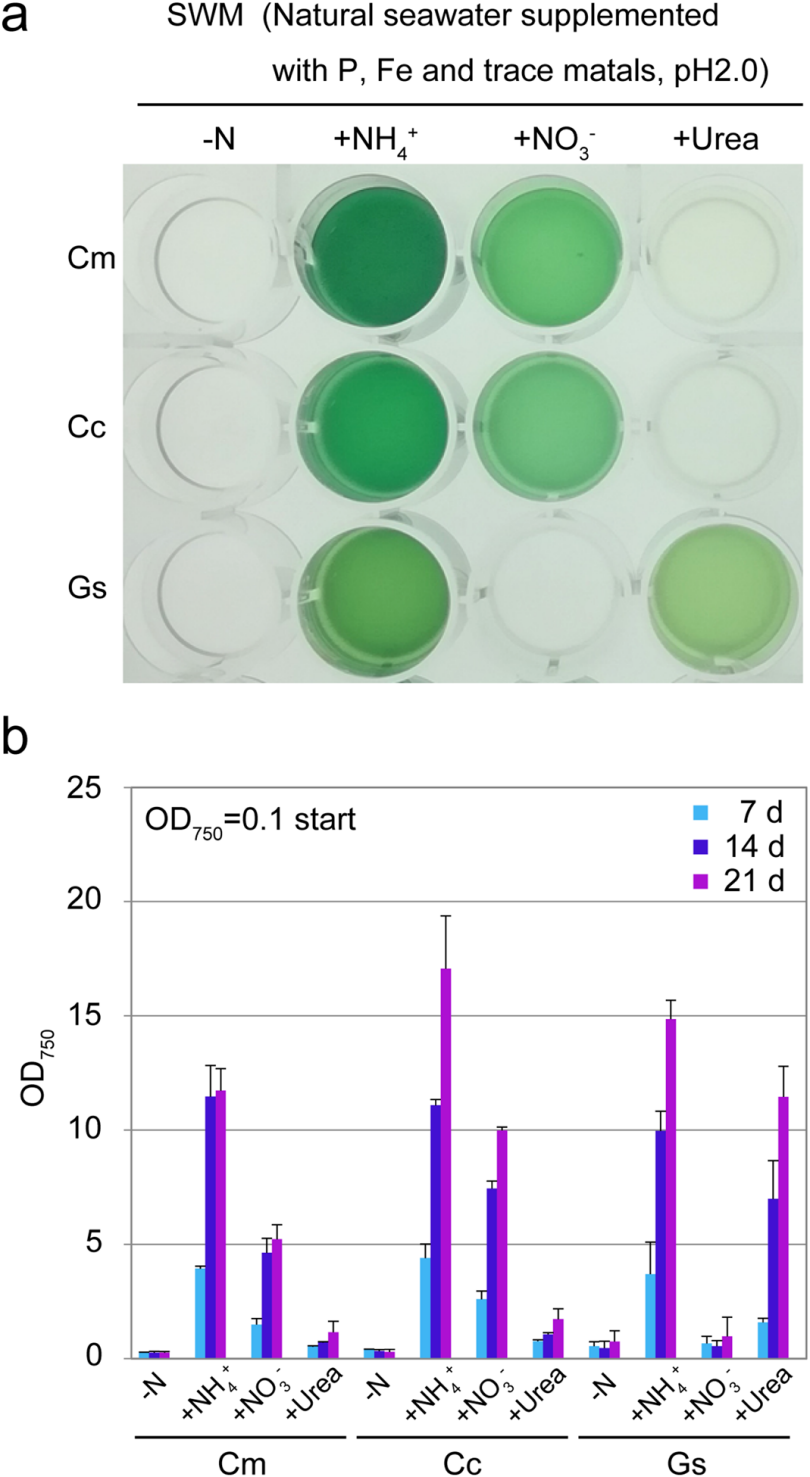
Growth of *C. merolae*, *Cy. caldarium*, *G. sulphuraria* cells in the seawater medium (SWM; the same as medium #15 in Fig. 2) supplemented with 20 mM (NH_4_)_2_SO_4_, 40 mM NaNO_3_, or 20 mM urea. (**a**) Cells cultured in MA with 0.3 M NaCl (OD_750_ of 1.0–2.0) were collected by centrifugation, resuspended in the respective media to give an OD_750_ of 0.1, and then cultured for 21 days. The image shows the cultures in the respective media 21 days after inoculation. (**b**) OD_750_ of cultures 7, 14, and 21 days after inoculation. Each data point represents the average and the error bar represents the standard deviation of three independent experiments. Cm, *C. merolae* 10D; Cc, *Cy. caldarium* RK-1; Gs, *G. sulphuraria* 074 W.

An inorganic nitrogen source is one of the most important nutrients that limit the rate of algal growth^24^. There are various forms of inorganic nitrogen sources, including nitrate, nitrite, ammonium, and urea, that are used by algae, but the forms that are used depend on the algal species^25^. To investigate the forms of inorganic nitrogen sources that support the growth of cyanidialean red algae in the seawater-based medium, the three algal species were cultured in SWM (natural seawater supplemented with KH_2_PO_4_, Fe-EDTA, and trace metals, at pH2.0) supplemented with ammonium (20 mM (NH_4_)_2_SO_4_), nitrate (40 mM NaNO_3_), or 20 mM urea (Fig. 3).

Similar to *C. merolae* (Fig. 2), *Cy. caldarium* and *G. sulphuraria* utilized the ammonium in SWM (Fig. 3). *C. merolae* and *Cy. caldarium* cells also used the nitrate in SWM, although their growth rates were lower than those in SWM supplemented with ammonium (Fig. 3). In contrast, *G. sulphuraria* could not utilize nitrate in SWM. This result is consistent with the previous report, in which *G. sulphuraria* did not grow in a synthetic inorganic medium that contained nitrate as the sole nitrogen source^19,26^. However, another study found that *G. sulphuraria* did utilize nitrate^27^. Thus, it is likely that the utilization of nitrate depends on the cultivation conditions and/or composition of media other than just the nitrogen source. Regarding urea, only *G. sulphuraria* grew in SWM supplemented with urea, although the growth rate was lower than that in SWM supplemented with ammonium (Fig. 3). In summary, the results showed that ammonium was the most favorable nitrogen source for all three species in the seawater-based medium (Fig. 3) as shown in a previous study based on cultivation in synthetic media^19^.

### Comparison of growth of cyanidialean red algae in the synthetic inorganic medium with or without 0.6 M NaCl, and the seawater-based medium

**Figure 4:**
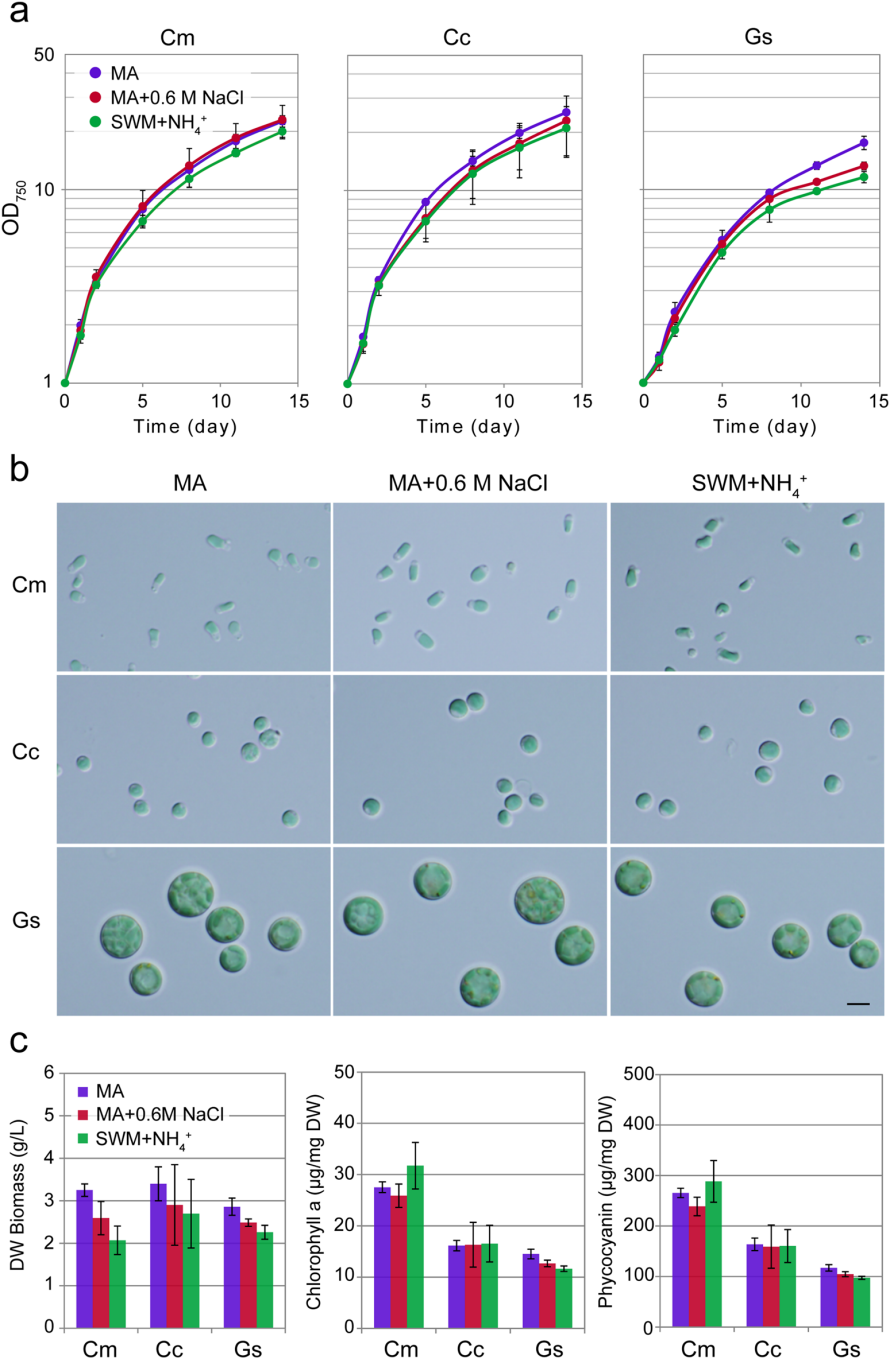
Growth of *C. merolae*, *Cy. caldarium,* and *G. sulphuraria* cells in MA, MA with 0.6 M NaCl, and SWM with ammonium. (**a**) Growth curves of *C. merolae*, *Cy. caldarium,* and *G. sulphuraria* in the respective media. Each data point represents the average and the error bar represents the standard deviation of three independent experiments. (**b**) Micrographs of cells that were cultured in the respective media 14 days after inoculation. Images were obtained by differential interference contrast microscopy (DIC). Scale bar = 5 μm. (**c**) Algal dry weight and chlorophyll *a* and phycocyanin contents per algal dry weight 14 days after inoculation. Each data point represents the average and the error bar represents the standard deviation of three independent experiments.

As described above, we succeeded in the cultivation of cyanidialean red algae in the natural seawater-based medium. We next compared the yield of the algae cultured in synthetic and seawater-based media. To this end, the three algal species grown in MA medium were transferred to a fresh MA medium, and those grown in MA with 0.3 M NaCl were transferred to MA with 0.6 M NaCl or SWM with ammonium. OD_750_ of all three species increased similarly, although the increase of *G. sulphuraria* was slightly compromised in the MA with 0.6 M NaCl or SWM compared with MA (Fig. 4a). Furthermore, there were no detectable differences in cellular morphology or color in all three species, regardless of the type of media (Fig. 4b). Consistent with this observation, the dry weight biomass of three algae grown in MA + 0.6 M NaCl or SWM + NH_4_^+^ was comparable to those in MA although that was slightly compromised in the presence of 0.6 M NaCl or seawater in *C. merolae* and *G. sulphuraria* culture (Fig. 4c). In addition, chlorophyll *a* and phycocyanin contents per cellular dry weight in SWM containing ammonium were comparable to those in MA and MA with 0.6 M NaCl (Fig. 4c).

The phycocyanin contents in *C. merolae*, *Cy. caldarium*, and *G. sulphuraria* cultured in SWM supplemented with ammonium were 288.6 ± 41.3, 160.6 ± 32.5, and 97.4 ± 2.9 μg/mg dry weight, respectively (Fig. 4c). These values are comparable to the amounts reported in the cyanobacterium *Spirulina platensis* (148.3 μg/mg dry weight), which is currently used for phycocyanin production for commercial uses^28^.

### Optimum and limit of pH for the growth of *C. merolae*

Many microalgae possess a rigid cell wall that requires mechanical processing to be disrupted to release the cellular contents. In contrast, *C. merolae* does not possess a rigid cell wall, which makes it easier to extract the cellular contents^21^. In addition, *C. merolae* is genetically tractable^21^*.* Thus, among cyanidialean red algae, *C. merolae* possesses a higher potential for use in several types of industrial fields than the others. Given these advantages of *C. merolae*, we further examined the optimal and limit of pH for *C. merolae* in the seawater-based medium. Generally, the uptake of ammonium ions by algae results in a pH drop, whereas that of nitrate leads to a pH increase, which often inhibits algal growth^29^. Thus, in many cases, algal cultivation requires chemicals to buffer the media during algal growth.

**Figure 5:**
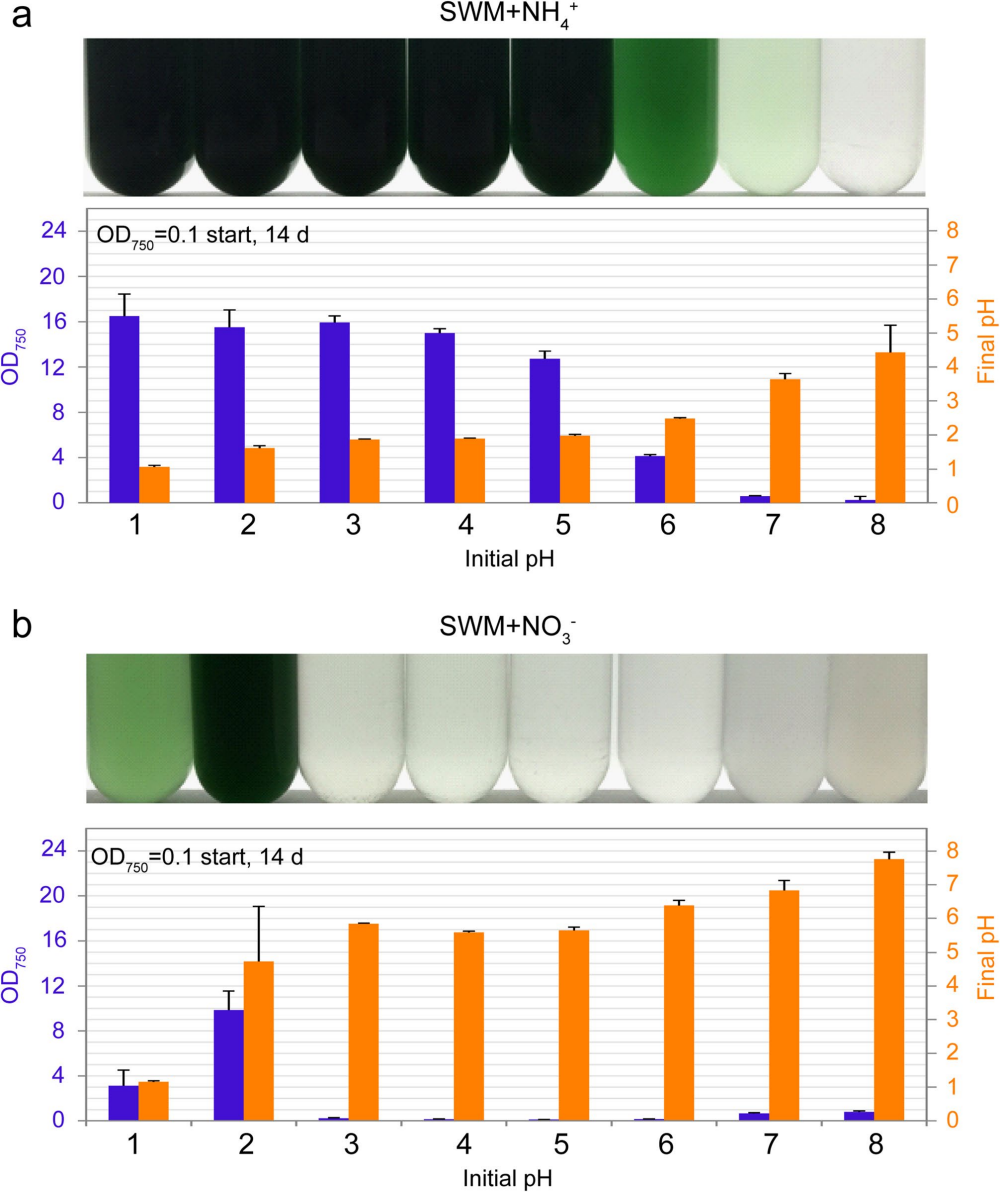
Growth of *C. merolae* in SWM supplemented ammonium or nitrate at different pH ranges. (**a**) Cells cultured in MA with 0.3 M NaCl (OD_750_ of 1.0–2.0) were collected by centrifugation, resuspended in SWM supplemented with ammonium (20 mM (NH_4_)_2_SO_4_) and at different pHs (from 1.0 to 8.0) to give an OD_750_ of 0.1, and then cultured for 14 days. The photograph shows the cultures 14 days after inoculation. The graph shows OD_750_ (blue bar) and final pH (yellow bar) of cultures 14 days after inoculation. Each data point represents the average and the error bar represents the standard deviation of three independent experiments. (**b**) The same as in panel a, except that cells were inoculated into SWM with nitrate (40 mM NaNO_3_) instead of SWM with ammonium.

To determine the optimal and limit of pH for *C. merolae* cultivation in the seawater-based medium, cells grown in MA supplemented with 0.3 M NaCl were transferred to ammonium- or nitrate-containing SWM at different pHs ranging from 1.0 to 8.0 and cultured (Fig. 5). When the cultures in SWM with ammonium were started at a pH of 1.0–8.0, the cells proliferated in the medium at start pH values from 1.0–7.0, and the media at pH 1.0–5.0 were the most suitable for growth (Fig. 5). When the initial pH was higher than 2.0, the pH value decreased during cultivation (Fig. 5). In contrast, when the culture in SWM with nitrate was started at a pH of 1.0–8.0, cells proliferated only in the medium at start pH values of 1.0 and 2.0. In these cases, the pH value increased during cultivation. The cellular growth rate in SWN with nitrate at any pH value was lower than that in SWN with ammonium (Fig. 5).

Although cultivation in SWM with ammonium at an initial pH value of > 2.0 decreased the pH of the medium, the pH values converged at 1.0–2.0 (Fig. 5a), which was known as the optimal value to promote *C. merolae* growth. Thus, the cultivation of *C. merolae* in SWM with ammonium does not require chemical buffers and pH controllers, which reduces the costs of cultivation.

### Semi-open cultivation of *C. merolae* in the seawater-based medium

**Figure 6:**
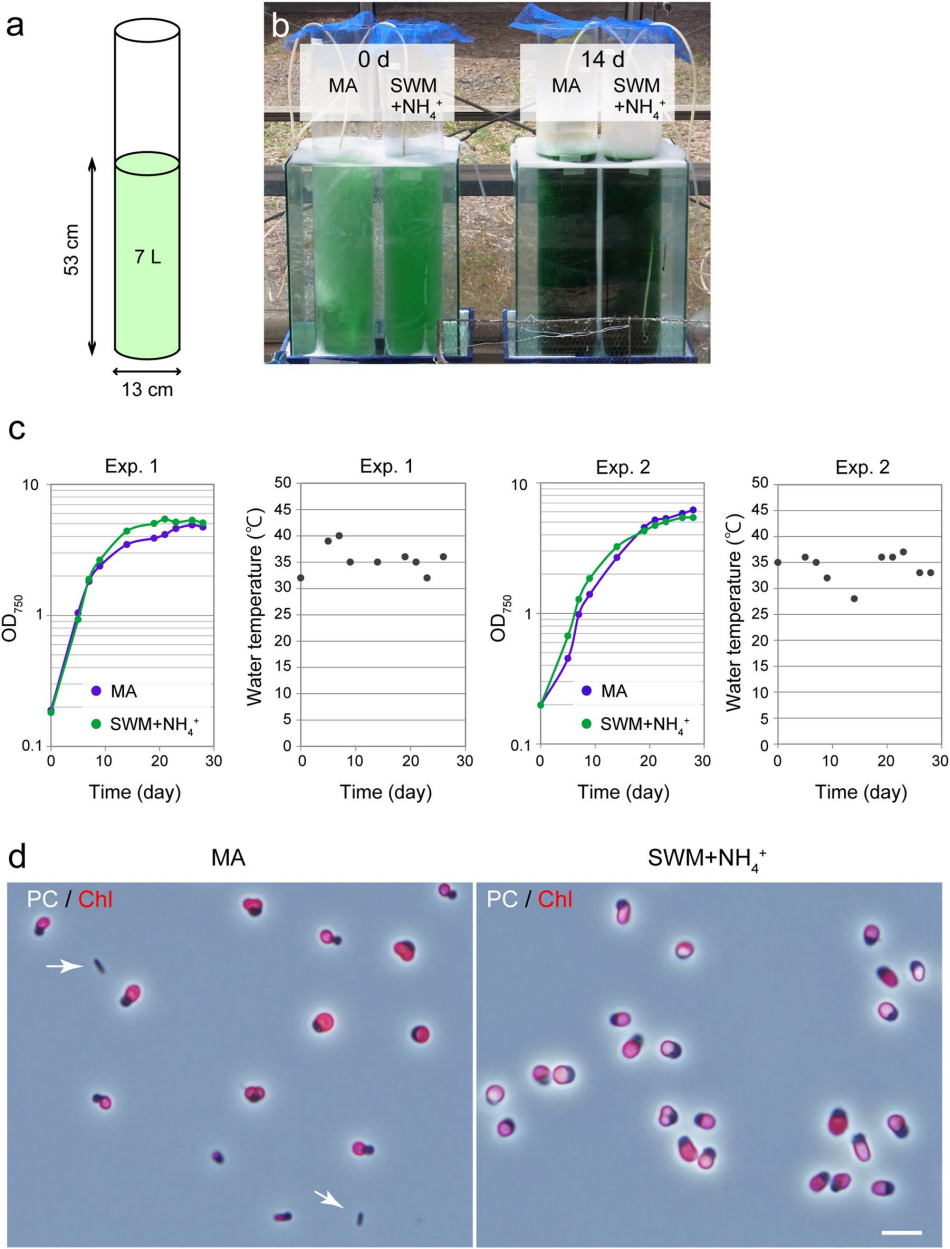
Outdoor cultivation of *C. merolae* in MA and SWM supplemented ammonium. A preculture in the laboratory in MA or SWM with ammonium was inoculated into 7 L of nonsterile MA or SWM with ammonium. The culture was 13 cm in diameter and 53 cm in height as shown in (**a**) to give an OD_750_ of 0.2. Cells were cultured for 28 days with aeration (7.5 L ambient air/min). The containers were open but the surface was covered with 4 mm mesh to avoid the contamination of relatively large materials, and placed into a water bath (without any temperature control) to moderate changes in culture temperature. The cultivation containers in the water bath were set inside a semi-open (one side was open) greenhouse without any temperature control. Experiment 1 was performed from July 31st to August 28th, 2019, and Experiment 2 was performed from August 14th to September 11th, 2019. In both cases, the highest intensity of sunlight was ~ 2,000 µmol m^–2^ s^–1^. (**b**) A photograph showing the cultures in MA and SWM supplemented with ammonium at day 0 for Experiment 1 and day 14 for Experiment 2. (**c**) Growth curves of *C. merolae* cultured in the respective media. Change in the temperature of the culture (temperature of the water in the water bath). (**d**) Micrographs of cells that were cultured in the respective media 14 days after inoculation. Images were obtained by phase contrast microscopy (PC), and the fluorescence images of chloroplasts were overlaid. Scale bar = 5 μm.

We finally tested whether the cultivation of *C. merolae* in SWM with NH_4_^+^ reduces the risk of microbial contamination in outdoor open cultivation as expected. To this end, we cultured *C. merolae* in SWM with ammonium in a semi-open greenhouse without temperature control (the side is not closed, but the greenhouse avoided inflow of rainwater). For comparison, the cells were also cultured in the synthetic MA medium without any NaCl supplement. The cells precultured in MA or SWM with ammonium in the laboratory were transferred to 7 L of nonsterile respective media in a cylindrical glass container without a lid, which was set in the greenhouse (Fig. 6a, b). The culture was performed twice from July to September 2019, in which the culture temperature was kept 28–40 °C (Fig. 6c) and the light intensity at noon was 300–2,000 μmol m^−2^ s^−1^ depending on the weather. In both trials, *C. merolae* cells in SWM with ammonium grew along a similar time course and to similar amounts compared with cells cultured in the synthetic MA medium (Fig. 6c). The final algal yields were 0.597/0.748 and 0.563/0.488 dry weight biomass g/L (experiment 1 / experiment 2) in MA and SWM + NH_4_^+^, respectively. These values were lower than those in laboratory condition (Fig. 4c). However, there is a room for improvement by reducing self-shading of algal cells for example by reducing the depth of the culture. The microscopic observation of cultures 14 days after inoculation showed that the culture in the freshwater MA medium was contaminated with bacteria in both trials (Fig. 6d). In contrast, no bacteria or organisms other than *C. merolae* were observed in the culture in SWM with ammonium in both trials (Fig. 6d). Thus, the combination of seawater and acidic conditions (SWM with ammonium), which do not exist in nature, reduces the risk of microbial contamination more efficiently than the acidic condition alone (MA).

### Usage of microalgal cultivation in acidified seawater

In this study, we performed the nonsterile open culture of *C. merolae* in a greenhouse to avoid the dilution of the medium by rainwater because the culture scale was relatively small (7 L). However, in a larger scale experiment, the inflow of rainwater or concentration of the medium by drying would probably not significantly affect the cellular growth of *C. merolae*. This is because *C. merolae* cells acclimated to 0.3 M NaCl and were able to grow in MA supplemented with 0–1.0 M NaCl (Fig. 1), which suggested they became tolerant to the sudden change of osmotic pressure and salinity.

In this study, the algae were cultured in the seawater-based medium with different intensities of light as follows. The stock culture was maintained at 20 μmol m^−2^ s^−1^. The static culture in 24-well culture plates (Figs. 2 and 3) were performed at 60 μmol m^−2^ s^−1^ because of the limitation of the growth chamber that we used. Other cultivations in laboratory (Fig. 5) were performed at 100 μmol m^−2^ s^−1^ which is the optimal intensity for *C. merolae*^30^. In addition, in the outdoor cultivation, the light intensity at noon was 300–2000 μmol m^−2^ s^−1^ depending on the weather. Despite the difference in the light intensity as above, there was slight difference in the algal growth between the synthetic medium and that with 0.6 M NaCl (Fig. 1b) or between the synthetic medium and the seawater-based medium (Fig. 4). Thus, the differences in light intensity did not affect the algal salinity tolerance.

In addition to *C. merolae*, we also found that *Cy. caldarium* and *G. sulphuraria* were able to grow in the seawater-based medium (Fig. 3). Among cyanidiales, *G. sulphuraria* is able to grow heterotrophically and mixotrophically in the presence of more than 50 different carbon sources in contrast to the obligate photoautotrophs *C. merolae* and *Cy. caldarium*^31^*.* In heterotrophic cultures, the yield of *G. sulphuraria* biomass is very high, and it was reported that a fed-batch culture produced 80–110 g/L biomass^32,33^. Given this high yield and heterotrophy, applications of this alga have been considered for phycocyanin production^34,35^, wastewater treatment^18^, and utilization of food waste^36^, although *G. sulphuraria* possess a rigid cell wall, unlike *C. merolae*. Although this remains to be tested, seawater will likely be applicable for the heterotrophic cultivation of *G. sulphuraria* to reduce the culture costs and contamination of other microorganisms.

In addition to cyanidialean red algae, acidophilic freshwater algae are distributed throughout different branches of eukaryotes, such as in green algae, stramenopiles, and euglenids. Thus, the identification of useful acidophilic algae and the application of seawater to their cultivations will also lead to biomass production by open pond culture systems with relatively low costs and contamination risks.

## Conclusion

We have developed a culture system in which acidophilic freshwater cyanidialean red algae grow in acidified natural seawater supplemented with inorganic nutrients. The utilization of seawater for cultivation reduces costs compared with the use of freshwater. In addition, we found that the cultivation does not require additional pH buffering chemicals and that the seawater-based open cultivation of acidophiles reduces the risks of contamination. The combination of seawater and highly acidophilic conditions, which do not exist in nature, will be useful for the open pond cultivation of acidophilic algae with little contamination of other organisms.

## Methods

### Algal strains

*Cyanidioschyzon merolae* 10D (NIES-3377), *Cyanidium caldarium* RK-1 (NIES-2137), and *Galdieria sulphuraria* 074W^37^ were used in this study. They were maintained in the inorganic M-Allen (MA) medium^22^ at pH 2.0 in Erlenmeyer flasks with gyration at 42 °C under continuous light (20 µmol m^–2^ s^–1^).

### Culture conditions for determining NaCl tolerance of the cyanidialean red algae

Different concentrations of NaCl (0–1000 mM) were supplemented to the MA medium. The three algal strains cultured in MA or MA supplemented with 0.3 M NaCl at 42 °C (OD_750_ of 1.0–2.0) were collected by centrifugation at 1500×*g* for 5 min and then gently resuspended into 1 mL of each medium in a 24-well culture plate to give an OD_750_ of 0.1. The cells were then cultured in an incubator with 2% CO_2_ at 42 °C under continuous light (60 μmol m^−2^ s^−1^) and without agitation for 7 days. OD_750_ was measured with a spectrophotometer (BioSpectrometer basic; Eppendorf, Hamburg, Germany).

### Examination of the effects of inorganic supplements on *C. merolae* growth in acidified seawater

Natural seawater (NAZEME 10; surface seawater collected from offshore of Izu peninsula, Shizuoka, Japan; Blue lab, Japan) was adjusted to a pH 2.0 with H_2_SO_4_, and then several combinations of 20 mM (NH_4_)_2_SO_4,_ 4 mM KH_2_PO_4_, Fe-EDTA (0.1 mM FeCl_3_, 0.075 mM EDTA·2Na), and the trace metal mix (18 μM MnCl_2_·4H_2_O, 1.5 μM ZnCl_2_, 3.2 μM Na_2_MoO_2_·2H_2_O, 0.34 μM CoCl_2_·6H_2_O, and 0.64 μM CuCl_2_)^22^ were added. The concentration of each inorganic supplement indicated above was the same as in the MA medium. *C. merolae* cultured in MA supplemented with 0.3 M NaCl at 42 °C (OD_750_ of 1.0–2.0) were collected by centrifugation at 1,500 × *g* for 5 min and then gently resuspended into 1 mL of each medium in a 24-well culture plate to give an OD_750_ of 0.1. The cells were then cultured in an incubator with 2% CO_2_ at 42 °C under continuous light (60 μmol m^−2^ s^−1^) and without agitation for 7 days.

### Examination of the utilization of different inorganic nitrogen sources by the cyanidialean red algae in the acidified seawater

Seawater medium (SWM), the natural seawater supplemented with KH_2_PO_4_, Fe-EDTA, and trace metals, was supplemented with either 20 mM (NH_4_)_2_SO_4_, 40 mM NaNO_3_, or 20 mM urea. The pH value was adjusted to 2.0 with H_2_SO_4_. The three algal strains cultured in MA with 0.3 M NaCl at 42 °C (OD_750_ of 1.0–2.0) were collected by centrifugation at 1,500 × *g* for 5 min and then gently resuspended into 1 mL of each medium in a 24-well culture plate to give an OD_750_ of 0.1. The cells were then cultured in an incubator with 2% CO_2_ at 42 °C under continuous light (60 μmol m^−2^ s^−1^) and without agitation.

### Comparison of the growth of the cyanidialean red algae in MA, MA with 0.6 M NaCl, and SWM with NH_4_^+^

The three algal strains cultured in MA or MA + 0.3 M NaCl at 42 °C (OD_750_ of 1.0–2.0) were collected by centrifugation at 1,500 × g for 5 min. The cells grown in MA were gently resuspended in 30 mL of MA in a 100-mL test tube to give an OD_750_ of 1.0. The cells grown in MA + 0.3 M NaCl were gently resuspended in 30 mL of MA with 0.6 M Nacl or SWM with NH_4_^+^ in a 100-mL test tube to give an OD_750_ of 1.0. The cells were then cultured at 42 °C under continuous light (100 μmol m^−2^ s^−1^) with aeration (0.3 L ambient air/min).

### Determination of the cellular dry weight and concentrations of chlorophyll *a* and phycocyanin

To determine the contents of chlorophyll *a* and phycocyanin, the absorbance of the cell culture medium was measured at wavelengths of 620 and 678 nm, respectively, using a spectrophotometer equipped with an integrating sphere (UV-2600; Shimazu, Kyoto, Japan). The chlorophyll *a* and phycocyanin contents were estimated according to the method^38^. To measure the dry weight, 10 mL of the cell culture was centrifuged using a pre-weighed 15-mL conical tube, and the supernatant was removed. The conical tube was dried overnight at 50 °C, and the cellular dry weight was measured on a microbalance.

### Microscopy

Samples were observed with a fluorescence microscope (BX51; Olympus, Tokyo, Japan) equipped with a digital camera (DP71; Olympus, Tokyo, Japan). Images were processed digitally with Photoshop software 2020 (https://www.adobe.com/in/products/photoshop.html version 21.1.1).

### Determination of the effect of different pH ranges on ***C. merolae*** culture in SWM with NH_4_^+^ or NO_3_^-^

SWM with NH_4_^+^ or NO_3_^-^ was adjusted to eight different pHs (from 1.0 to 8.0) with HCl or NaOH. Media at a pH of 8.0 produced precipitates but these were used as they were. *C. merolae* cells cultured in MA with 0.3 M NaCl medium at 42 °C (OD_750_ of 1.0–2.0) were collected by centrifugation at 1,500 × g for 5 min and then gently resuspended in 30 mL of each medium in a 100-mL test tube to give an OD_750_ of 0.1. The cells were cultured at 42 °C under continuous light (100 μmol m^−2^ s^−1^) with aeration (0.3 L ambient air/min). The pH of the respective cultures was examined 14 days after inoculation.

### Outdoor semi-open cultivation of *C. merolae*

*C. merolae* grown in MA or SWM with NH_4_^+^ at 42 °C under continuous light (100 μmol m^−2^ s^−1^) with aeration (1 L ambient air/min) were transferred into 7 L of medium in a cylindrical glass container to give an OD_750_ of 0.2. The containers were open, but the surface was covered with 4 mm mesh to prevent the contamination by relatively large materials. The containers were placed into a water bath (without any temperature control) to moderate changes in culture temperature. The cultivation containers in the water bath were set inside a semi-open (one side was open) greenhouse without any temperature control, and the cells were cultured with aeration (7.5 L ambient air/min)*.* The experiments were conducted from July to September 2019 and were independently repeated twice on different days.
